# Racial-Ethnic Disparities in Access to Preventive Services Among Privately Insured Adults With Disabilities

**DOI:** 10.1093/geroni/igab046.2097

**Published:** 2021-12-17

**Authors:** Elham Mahmoudi, lauren Groskaufmanis, Neil Kamdar, Anam Khan, Mark Peterson

**Affiliations:** 1 University of Michigan, Commerce Township, Michigan, United States; 2 University of Michigan, Ann Arbor, Michigan, United States; 3 University of Michigan School of Public Health, Ann Arbor, Michigan, United States; 4 University of Michigan, University of Michigan, Michigan, United States

## Abstract

Introduction: Cerebral palsy (CP) and spina bifida (SB) are congenital disabilities. Due to life-long disability, adults with CP/SB are with greater needs for preventative care. Little is known about racial/ethnic disparities in use of preventative services in this population. Our objective was to examine racial/ethnic disparities in use of preventative care. Methods: Using 2007-2017 private claims data, we identified White, Black, and Hispanic adults (18+) with CP/SB [n=11,635; White=8,935; Black=1,457; Hispanic=1,243)]. We quantified the National Institute of Medicine (NAM) definition of disparity by matching health related variables (age, sex, comorbid conditions, and Elixhauser index) between Whites and each minority subpopulation. Generalized estimating equations were used and all models were adjusted for age, sex, comorbidities, income, education, and U.S. Census divisions. Outcomes of interest were: (1) any office visit; (2) any physical therapy/ occupational therapy (PT/OT); (3) annual wellness visit; (4) bone density screening; (5) cholesterol screening; (6) diabetes screening. Results: Rate of recommended services for all adults with CP/SB were low and no significant results were found for most preventative services across race/ethnicity. Compared with Whites, Hispanics had lower odds of annual wellness visit (OR: 0.71; 95% CI: 0.53, 0.96) but higher odds of diabetes screening (OR: 1.48; 95% CI: 1.13, 1.93). Blacks had lower odds of bone density screening (OR: 0.54; 95% CI: 0.31-0.95), and annual wellness visit (OR: 0.50; 95% CI: 0.24-1.00). Conclusions: There were no substantial racial/ethnic disparities in use of preventive services among privately insured adults with CP/SB who had a higher-than-average income and education level.

